# Meta-Analysis of Maternal and Neonatal Outcomes Associated with the Use of Insulin Glargine versus NPH Insulin during Pregnancy

**DOI:** 10.1155/2012/649070

**Published:** 2012-05-16

**Authors:** Jacques Lepercq, Jay Lin, Gillian C. Hall, Edward Wang, Marie-Paule Dain, Matthew C. Riddle, Philip D. Home

**Affiliations:** ^1^Service de Gynécologie-Obstétrique, Hôpital Cochin-Saint Vincent de Paul, Cedex 14, 75674 Paris, France; ^2^Outcomes Research, Novosys Health, Flemington, NJ 08822, USA; ^3^Grimsdyke House, EN5 4ND London, UK; ^4^Diabetes-Metabolism Franchise, Sanofi-Aventis France, Cedex 14, 75159 Paris, France; ^5^Department of Medicine, Division of Endocrinology, Diabetes & Clinical Nutrition, Oregon Health and Science University, Portland, OR 97239, USA; ^6^Institute of Cellular Medicine–Diabetes, Newcastle University, Newcastle upon Tyne NE2 4HH, UK

## Abstract

As glargine, an analog of human insulin, is increasingly used during pregnancy, a meta-analysis assessed its safety in this population. A systematic literature search identified studies of gestational or pregestational diabetes comparing use of insulin glargine with human NPH insulin, with at least 15 women in both arms. Data was extracted for maternal outcomes (weight at delivery, weight gain, 1st/3rd trimester HbA_1c_, severe hypoglycemia, gestation/new-onset hypertension, preeclampsia, and cesarean section) and neonatal outcomes (congenital malformations, gestational age at delivery, birth weight, macrosomia, LGA, 5 minute Apgar score >7, NICU admissions, respiratory distress syndrome, neonatal hypoglycemia, and hyperbilirubinemia). Relative risk ratios and weighted mean differences were determined using a random effect model. Eight studies of women using glargine (331) or NPH (371) were analyzed. No significant differences in the efficacy and safety-related outcomes were found between glargine and NPH use during pregnancy.

## 1. Introduction

An estimated 4% of pregnancies in the United States are complicated by diabetes [1]. Whether due to preexisting type 1 or type 2 diabetes mellitus (pregestational) or diabetes that developed during pregnancy (gestational), hyperglycemia during pregnancy is associated with increased risk of various maternal and fetal complications. Subclinical increases in fasting blood glucose levels as little as 6.9 mg/dL and elevated postprandial plasma glucose levels have been associated with a greater risk of developing both maternal and fetal complications [2]. Women with pregestational diabetes may experience worsening of retinopathy or nephropathy [3], and both pregestational and gestational diabetes are associated with increased risk of hypertension, preeclampsia, and delivery by cesarean section [4–13]. Fetal complications include congenital malformations, premature delivery, perinatal death, macrosomia and traumatic delivery, neonatal hypoglycemia or respiratory distress, admission to a neonatal intensive care unit, and risk of developing obesity or diabetes later in life [4, 9–25]. Because many of these problems may be reduced by improved glycemic control [26], efforts to maintain nearly normal levels of glucose are recommended during pregnancy associated with diabetes, frequently requiring the use of insulin.

The insulin preparations most widely used in this setting have been neutral protamine hagedorn (NPH) human insulin and short-acting human insulin, or insulin analogs. American Diabetes Association (ADA) guidelines recommend that patients using basal insulin analogs be transitioned to NPH insulin, preferably prior to pregnancy [27]. Clinical trials have shown that, in comparison to NPH, the basal insulin glargine can, under appropriate circumstances, improve glycemic control and reduce the frequency of hypoglycemia and thus may be beneficial in pregnancies associated with diabetes [28, 29]. However, the safety of using insulin glargine during pregnancy has not been fully demonstrated, and concern about abnormal binding of this analog to the insulin-like growth factor 1 (IGF-1) receptor has been voiced [30–32]. For this reason, we performed a literature search and meta-analysis to examine published experience with the use of insulin glargine, assessing both maternal and fetal outcomes.

## 2. Methods

### 2.1. Identification of Studies

Studies published by 30 January 2011 were identified by a systematic literature search in MEDLINE, EMBASE, and the Cochrane Central Register for Controlled Trials database. The search was executed with no language restrictions and using pairwise combinations “insulin glargine” or Lantus with the following terms: pregnancy, pregnant, neonatal, fetal, foetal, perinatal, and maternal. Hand searching of the reference lists of the retrieved articles and relevant reviews was performed. The registry at http://www.clinicaltrials.gov/ was searched to identify unpublished clinical trial data regarding insulin glargine use during pregnancy reported by the cutoff date. Unpublished studies reported in abstracts from conferences were excluded from the study. Two investigators independently screened the title and abstract of each reference identified by the search and applied the following inclusion criteria with any differences in opinion resolved by a third party. The inclusion criteria consisted of (1) study type—retrospective or prospective observational case-control study, cohort study, or randomized controlled trial; (2) participants—pregnant women with pregestational and/or gestational diabetes; (3) interventions—insulin glargine and NPH insulin; (4) study size—studies with ≥15 women per arm. The full articles of those studies that satisfied the inclusion criteria were retrieved and assessed independently with the final eligibility of studies decided by consensus. The quality of the reporting of studies to be included in the meta-analysis was assessed using the Strengthening of the Reporting of Observational Studies in Epidemiology (STROBE) statement guidelines [33].

### 2.2. Data Extraction

Data extraction forms were used to obtain the data from each study included in the meta-analysis. Data was extracted for study subjects treated with either NPH or insulin glargine; study “control” subjects who did not have diabetes or did not receive basal insulin therapy were not included in the meta-analysis. The maternal baseline characteristics extracted included: maternal age, duration of diabetes, prepregnancy weight, and prepregnancy body mass index (BMI). The maternal outcomes assessed were weight at delivery, weight gain, 1st and 3rd trimester HbA_1c_ levels, episodes of severe hypoglycemia, gestational/new-onset hypertension, preeclampsia, and cesarean section. Neonatal outcomes were gestational age at delivery, birth weight, neonatal ICU (NICU)admission, 5 minute Apgar score <7, macrosomia (birth weight >4000 g), large for gestational age (birth weight >90th percentile for their gestational age, LGA), congenital malformations, respiratory distress syndrome, neonatal hypoglycemia, and hyperbilirubinemia.

### 2.3. Assessment of Heterogeneity

Heterogeneity between trials was assessed by the *χ* ^2^-test. Quantification of heterogeneity was also examined with *I* ^2^, which measures the degree of total variation across studies due to heterogeneity and can be used to judge the consistency of evidence. Higher *I* ^2^ values correspond to increasing heterogeneity [34].

### 2.4. Data Synthesis

In the event that a study stratified its participants by pregestational/gestational diabetes status or by diabetes type prior to analysis, the outcomes for each cohort were combined to create a single cohort result. For continuous data, the mean and standard deviation of the combined cohort were calculated from the separate cohort results using standard formulas. Dichotomous data presented as percentages were converted to counts and combined. All meta-analysis outcomes were assessed using a random effects model which incorporated the impact of heterogeneity in the analysis. Weighted mean differences (WMDs) with 95% confidence intervals (CIs) were determined for continuous data using the inverse variance method. Mantel-Haenszel odds ratios (OR) with 95% confidence intervals were determined for dichotomous data. All analyses were performed using Review Manager (RevMan, version 5.1, Copenhagen: Cochrane Collaboration). In addition, all forest plots were generated by RevMan.

## 3. Results

### 3.1. Description of Studies

**Figure 1:**
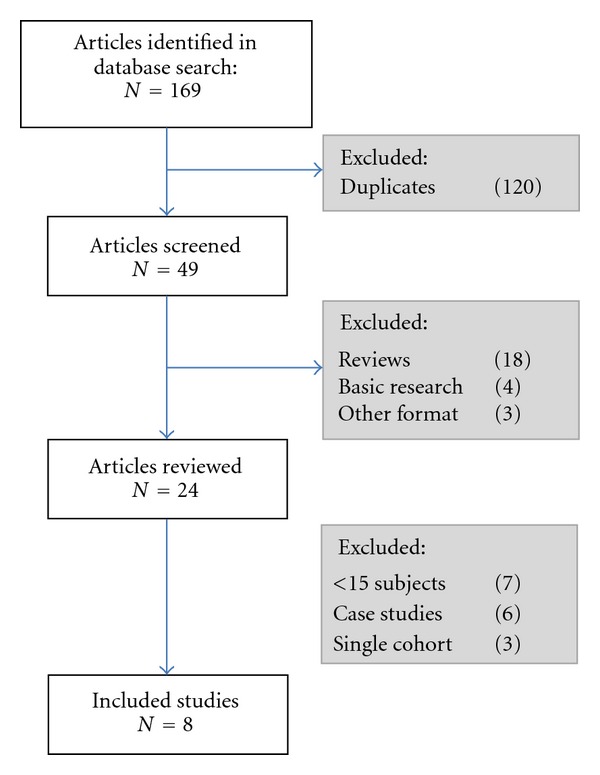
Study flow.

The initial literature search identified 49 articles; no further relevant studies or clinical data were retrieved from the searches of the Cochrane database or the clinical trial registry (Figure 1). Twenty-five of these articles did not contain human data and were excluded. An additional thirteen studies were excluded due to an insufficient number of participants; six articles were case studies, and seven were reports regarding the outcomes of four to thirteen subjects. Three studies were excluded due to the absence of an appropriate basal insulin comparison group. Eight studies satisfied the inclusion criteria and, after confirming adherence to STROBE guidelines, were included in the meta-analysis (Table 1) [35–42]. When combined, the eight observational cohort studies consisted of a total of 702 women with pregestational and/or gestational diabetes, of whom 331 received insulin glargine and 371 received NPH insulin. Five of the studies examined women with both pregestational and gestational diabetes [36, 37, 39, 41, 42]. Only two of the five studies specified the type of pregestational diabetes [41, 42]. The remaining three studies included only women with pregestational type 1 diabetes [35, 38, 40].

### 3.2. Maternal Baseline Characteristics

Baseline maternal characteristics are reported in Table 2. Information on each maternal baseline characteristic was reported in three to seven of the eight studies (Table 2). Overall, there was no difference in maternal age, body weight, or body mass index between the women using insulin glargine and those using NPH insulin, while the duration of diabetes was longer among insulin glargine users. Although women using insulin glargine were slightly younger compared to NPH insulin users, the difference was not statistically significant [35–39, 41, 42]. Similarly, while the mean prepregnancy weight of glargine users was 1.53 kg greater that of NPH users [37–39, 41] whose mean prepregnancy BMI was 0.46 kg/m^2^ greater than that of glargine users [35, 36, 41], weight and BMI were comparable between insulin groups in all studies and the overall mean differences were not statistically significant (Table 2).

Duration of diabetes was reported in seven studies [35, 36, 38–42]. While duration of diabetes was longer in glargine users (Table 2), there was considerable variation between the studies (*I* ^2^ = 48%). This may be explained by the combination of women with type 1 and type 2 diabetes into a single diabetes cohort. Subanalysis of five studies that reported the duration of type 1 diabetes revealed a more homogeneous population (*I* ^2^ = 6%), but glargine users still had longer duration of diabetes (Table 2, *P* = 0.0001) [35, 38, 40, 41]. In contrast, the three studies reporting the duration of pregestational diabetes without distinction between type of diabetes had a high degree of variation (*I* ^2^ = 71%) [36, 39, 42]. When standardized weighted mean differences were utilized to compensate for the variation in the duration of diabetes measurements that is inherent to the type of diabetes (i.e., due to earlier onset, patients with type 1 diabetes are expected to have a longer duration of diabetes in comparison to patients with type 2 diabetes), the mean difference between insulin glargine and NPH users was no longer statistically significant (0.38 years (−0.11 to 0.88)).

Eight studies reported on difference in maternal age between insulins, but only seven studies reported the mean maternal age [35–39, 41, 42]. When combined, there was no significant difference in maternal age between insulin glargine and NPH users (Table 2). Although six studies individually reported no difference in maternal age between glargine and NPH users, in one study, women with type 1 pregestational diabetes that were treated with insulin glargine were significantly younger than those that were treated with NPH [41]. This observation may explain some of the heterogeneity between the trials (*I* ^2^ = 57%).

### 3.3. Maternal Outcomes

**Figure 2:**
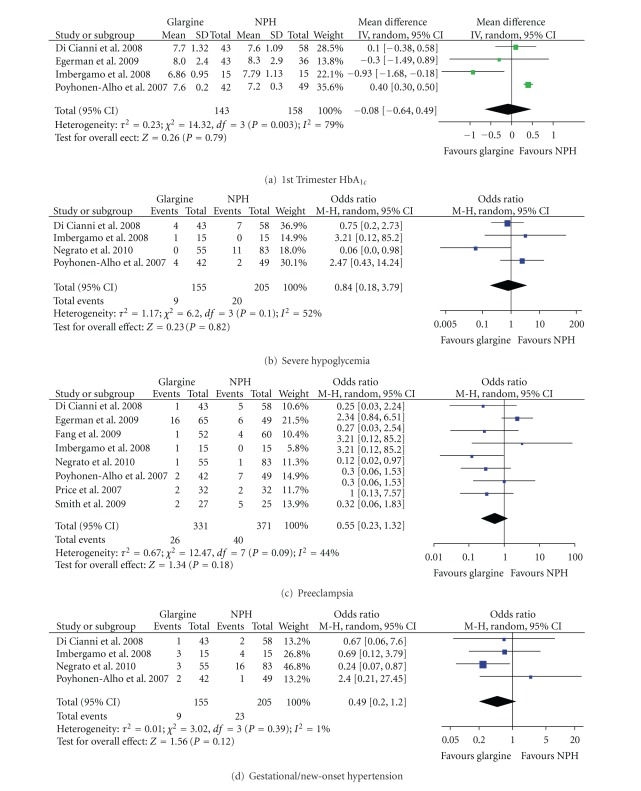
Meta-analysis results for maternal outcomes.

The meta-analysis included the data that was available for each maternal outcome in four to eight of the identified studies (Table 3). Overall, there was no significant difference in any maternal outcome between Glargine and NPH users (Table 3 and Figure 2). Additionally, third trimester HbA_1c_ [35–37, 39–42], maternal weight [36, 38, 39, 41], and rate of cesarean section at delivery [34–36, 38, 39, 41] did not differ significantly between insulins in any individual study.

Weight gain during pregnancy was reported in five studies [35–39]. Four studies individually reported that weight gain was comparable between insulins while one study reported lower weight gain among glargine users (6.7 versus 11.4 kg; *P* < 0.01) [41]. There was no difference in weight gain in the meta-analysis. HbA_1c_ levels measured at any time during the first trimester were reported in four studies (Figure 2(a)) [35, 36, 38, 40]. Of the three studies which investigated women with type 1 diabetes, one found that glargine use was associated with lower 1st trimester HbA_1c_ levels (6.9 versus 7.8%; *P* = 0.04) [38]. There was considerable variation in HbA_1c_ levels between the studies (*I* ^2^ = 79%) that could not be explained by the type of diabetes.

Although six studies reported hypoglycemia data, only three explicitly defined severe maternal hypoglycemia as an episode requiring the assistance of another person [35, 39, 40]. A further study was included as it used a classification of mild and severe hypoglycemia [38]. While there was no overall difference in the prevalence of severe hypoglycemia with glargine or NPH use (Table 3, Figure 2(b)), one study found that the use of NPH was associated with a higher incidence of severe hypoglycemia among women with pregestational diabetes (27% versus 0%; *P* < 0.0001) [39]. There was substantial heterogeneity between the studies (*I* ^2^ = 52%) not explained by type of diabetes.

The incidence of preeclampsia was reported in all eight studies (Figure 2(c)). There was sizable variation between the studies (*I* ^2^ = 44%) that could be explained by type of diabetes. Subanalysis of those studies investigating pregnant women with type 1 diabetes resolved the heterogeneity between the studies (*I* ^2^ = 0%) but did not result in a significant difference in preeclampsia (OR 0.39 (0.12 to 1.32); *P* = 0.13) [35, 38, 40]. One study reported that the incidence of preeclampsia was higher in pregestational women using NPH compared to those using insulin glargine (19% versus 0%; *P* < 0.0001) [39].

The incidence of gestational hypertension was reported in four studies (Figure 2(d)) [35, 38–40]. While there was little heterogeneity between the studies (*I* ^2^ = 1%), in one study, the use of NPH by women with gestational diabetes was associated with an increased incidence of gestational hypertension compared to insulin glargine (18% versus 2.5%; *P* < 0.0001) [39].

### 3.4. Neonatal Outcomes

**Figure 3:**
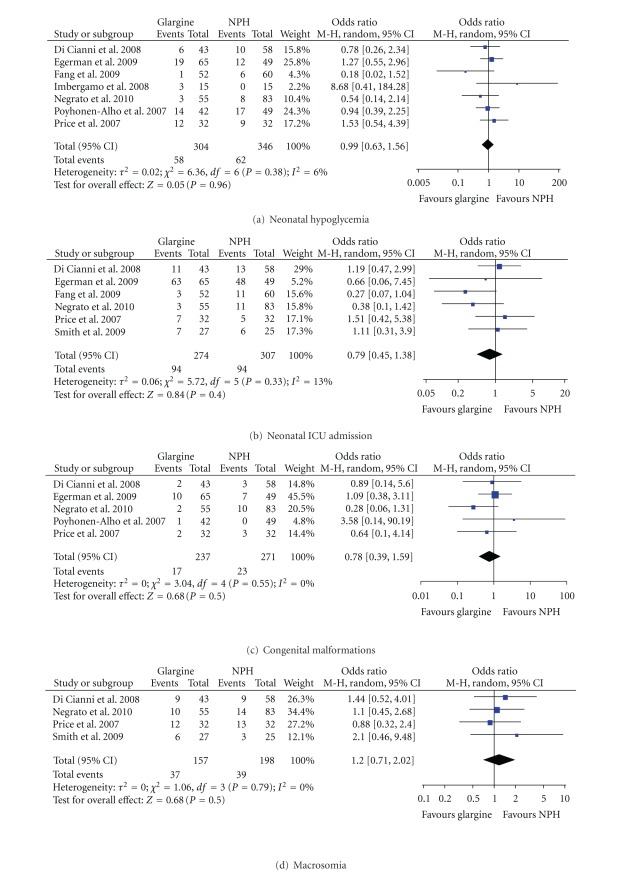
Meta-analysis results for neonatal outcomes.

Seven of the eight studies reported gestational age at delivery [35–39, 41, 42], birth weight [36–42], and neonatal hypoglycemia [35–41]. When the studies were analyzed, there were no differences between women that used glargine or NPH for these outcomes (Table 4, Figure 3(a)). Within the individual studies, there were also no differences except for one study which reported that NPH was associated with a significantly higher incidence of neonatal hypoglycemia (25% versus 0%; *P* = 0.01) [37].

Six studies reported the incidence of NICU admissions [35–37, 39, 41, 42], respiratory distress syndrome [36–41], and hyperbilirubinemia [35–40]; overall there was no difference between insulins (Table 4, Figure 3(b)). However, one study reported that the use of NPH during pregnancy was associated with an increased incidence of NICU admissions among the offspring of women with pregestational diabetes (16% versus 5.5%; *P* = 0.02) [39], and two studies reported that NPH was associated with an increased incidence of hyperbilirubinemia among the offspring of women with either pregestational or gestational diabetes (31.3% versus 8.3%; *P* = 0.05) [37] as well as the offspring of gestational diabetic pregnancies (9% versus 0%; *P* < 0.01) [39].

Five studies reported the incidence of congenital malformations [35, 36, 39–41]. While the meta-analysis study result was not statistically significant (Table 4, Figure 3(c)), one of the studies associated NPH use with a significant increase in the incidence of birth defects among the children of women with gestational diabetes when compared to insulin glargine use (13% versus 2.5%; *P* = 0.016) [39].

The incidence of macrosomia [35, 39, 41, 42], LGA infants [35, 37–39], and 5-minute Apgar scores <7 [37–39, 42] were each reported in four of the eight studies. There were no significant differences between the use of glargine and NPH among the combined or individual studies (Table 4, Figure 3(d)) with the exception of one study which found that the use of NPH was associated with a significantly increased incidence of LGA among the infants of women with pregestational diabetes (50% versus 18.9%; *P* = 0.04) [37].

Heterogeneity between the studies assessed for each of the neonatal outcomes was generally low, with the exception of the gestational age at delivery (*I* ^2^ = 71%) and the incidence of hyperbilirubinemia (*I* ^2^ = 44%). While the heterogeneity of the studies reporting the gestational age at delivery cannot be explained by the type of diabetes present during pregnancy, subanalysis of the type 1 pregestational diabetic women with hyperbilirubinemic children results in a homogenous population (*I* ^2^ = 0%), suggesting that the heterogeneity is due to combining the pregestational and gestational populations.

## 4. Summary

In this meta-analysis of eight observational studies, no significant increased risk associated with the use of insulin glargine compared with NPH insulin was discerned for any of the maternal or neonatal outcomes reported. With regard to the safety of insulin glargine use during pregnancy, in comparison to NPH insulin, there was no increased risk to the mother for weight gain, severe hypoglycemia, gestational/new-onset hypertension, preeclampsia, or cesarean section. While individual studies did inconsistently report differences, many of the individual findings were favorable to insulin glargine. Glycemic control as measured by first and third trimester HbA_1c_ was not different between the pregnant women using insulin glargine and those using NPH insulin.

## 5. Discussion

Few studies were identified which have addressed the safety of insulin glargine versus NPH use during pregnancy, and many of those identified were conducted with small study populations. The current meta-analysis of these published studies found no significant differences in maternal or fetal health outcomes or complications associated with the use of insulin glargine in comparison to NPH.

The neonatal outcomes of this study are in accordance with those reported in a recent meta-analysis of the neonatal safety of insulin glargine use during pregnancy [43]. While the eight studies identified in this meta-analysis are the same as those identified and analyzed in the previous study, it focused primarily on neonatal outcomes and no meta-analyses were performed on any of the maternal outcomes reported in the studies. Similar to the current study, there were no significant differences between the use of either insulin glargine or NPH with regard to adverse neonatal outcomes. Divergent data extraction methods resulted in the selection of slightly different study subject numbers or adverse event numbers for analysis. For example, among the 304 insulin glargine patients and 346 NPH patients with neonatal hypoglycemia data, the Pollex study identified 57 women treated with insulin glargine and 66 women treated with NPH whose offspring were hypoglycemic. In contrast, the current study identified 58 women treated with insulin glargine and 62 women treated with NPH. Meta-analysis of the neonatal hypoglycemia outcomes resulted in an odds ratios of 0.94 (0.64–1.39) for the Pollex study and 0.99 (0.63–1.56) for the current study; both differences were not statistically significant. Different statistical algorithms may also account for the variation between the results of the two studies. Analysis of the prevalence of respiratory distress syndrome utilized the same raw data but resulted in slightly lower but statistically nonsignificant odds ratios in the Pollex study (1.53 (0.82 to 2.85) versus 1.62 (0.82 to 3.21)). While the results are not identical, the corresponding neonatal outcomes in the current study are comparable with the previous report.

The present analysis demonstrated similarly equivalent findings between treatment with insulin glargine and NPH insulin for maternal outcomes, which were not addressed in the Pollex study. One aspect of the maternal findings of the present analysis requires further comment; women using insulin glargine had longer duration of diabetes than those using NPH insulin. Although the weighted mean difference in the duration of diabetes was significant, there was substantial heterogeneity between the studies. Subanalysis of the studies based on type of diabetes suggests that the heterogeneity was due to the combination of patients with type 1 and type 2 diabetes into a single cohort. The implications of this finding are uncertain. It is possible that the longer duration of type 1 diabetes among women using insulin glargine was due to women switching to the newer insulin glargine from established insulin regimens in order to achieve better glycemic control than that provided by their prior insulin therapy.

The finding in this analysis that maternal and fetal complications did not differ between women using insulin glargine and those using NPH insulin is consistent with recent findings regarding the metabolism and actions of insulin glargine. Much of the concern about insulin glargine stemmed from *in vitro* experiments exposing human osteosarcoma cells (Saos/B10) to insulin glargine which revealed a 6.5-fold higher affinity for the IGF-1 receptor and an 8-fold greater mitogenic action in comparison to insulin [31]. However, experiments using other cell lines and, in particular, human skeletal muscle cells, coronary artery cells, blood vessel endothelium cells, normal epithelial breast, and cancer cell lines have suggested a mitogenic and metabolic potency of insulin glargine similar to that of human insulin [30, 44–46]. Moreover, recent *in vivo* studies have shown that insulin glargine is converted into two metabolites which are metabolically active and may account for most of the action of insulin glargine, but which have an affinity for the IFG-1 receptor that is comparable to that of human insulin [47]. These results suggest that insulin glargine is unlikely to adversely affect fetal development [48, 49].

This meta-analysis has a number of limitations. It was based on data from observational studies and consequently, the participants were not randomized and there were insufficient data in the papers to allow statistical adjustment of the combined comparisons. While there was no significant difference in all but one of the baseline characteristics, the confidence intervals were often wide and not all studies reported each baseline characteristic. For example, only three of the eight studies utilized in the meta-analysis reported the BMI of study participants. Of those studies, the mean BMI of the women in two of the studies indicate that a significant number of them are obese. Studies have found that, similar to diabetes, obesity is associated with an increased risk of the same adverse outcomes including gestational hypertension, preeclampsia, cesarean section, stillbirth, congenital malformations, and macrosomia [50, 51]. In addition, there may be confounding due to unmeasured characteristics such as socioeconomic status and race. There was little racial/ethnic background information available for the participants of the study. This could be of importance as certain ethnic backgrounds may predispose subjects to certain adverse outcomes, confounding the results. A recent literature review found that ninety-three of the included 106 studies reported at least one significant association between socioeconomic measures and birth outcomes among the overall study population or within a racial or ethnic subgroup [52]. Maternal complications are also impacted by socioeconomic status and race; several studies have found that low socioeconomic status and nonwhite races are substantial risk factors for preeclampsia [53–55]. All but one of the studies was retrospective in design; data may not have been available for collection, limiting the scope of the study. Retrospective analysis also allowed for the selection of patients that had continuous insulin glargine or NPH treatment throughout pregnancy, precluding study of the impact of switching basal insulin use during pregnancy; only one study reported that a single patient was switched from NPH to insulin glargine during pregnancy [40]. The only observational prospective study included in the meta-analysis did not report any changes in basal insulin use [39]. Another limitation to the meta-analysis was the study population available for investigation. During the identification of studies to be included in the meta-analysis, few studies contained a sufficient number of patients to be included in the analysis; many of the excluded studies reported data for fewer than fifteen patients in total and often had only a single treatment arm. Among the eight studies included in the meta-analysis, only four contained data for more than one hundred patients among the combined treatment arms. The number of participants within each treatment arm was as low as fifteen patients per study, hindering statistical comparison between groups. While there were no statistically significant outcomes to report in this meta-analysis, the combined analysis of the individual study populations was underpowered due to the limited sample sizes. Consequently, the risk of rarer complications of pregnancy associated with diabetes such as worsening of preexisting maternal retinopathy or nephropathy, or occurrence of polyhydramnios and fetal mortality associated with diabetic ketoacidosis, cannot be assessed. An additional limitation to the study was the grouping of type 1 and type 2 diabetes patients into a single pregestational diabetes cohort. Only one of the studies included in the meta-analysis reported findings for all outcomes separately for each type of pregestational diabetes [42]. Moreover, only a subset of the studies compared outcomes between patients with pregestational and gestational diabetes [37, 39, 41]. Finally, exposure to insulin glargine or NPH during the 1st trimester was limited to the five studies that focused on pregestational diabetes.

## 6. Conclusions

The results of this meta-analysis did not show differences in maternal or neonatal outcomes in women with diabetes who were treated with glargine compared to NPH. Because the quantitative estimates of difference for individual measures had large uncertainties, accumulation of more data is warranted, preferably by conducting randomized controlled trials or prospective observational studies in which participants are followed for longer periods of time.

## Figures and Tables

**Table 1:** Characteristics of the eight study reports included in this meta-analysis.

Reference number	Author (Year)	Type of study	Time period	Number of women	Cohorts (*n*)	Type of diabetes	Comparison(s)
[35]	Di Cianni et al. (2008)	Retrospective	to December 31, 2006	101	Glargine (43) NPH (58)	Type 1 DM	Continuous glargine use throughout pregnancy versus cessation of glargine during pregnancy (replaced w/NPH)
[36]	Egerman et al. (2009)	Retrospective	January 2004 to August 2006	114	Glargine (65) NPH (49)	Pregestational Gestational	Insulin glargine versus NPH
[37]	Fang et al. (2009)	Retrospective	January 2003 to April 2008	112	Pregestational: glargine (37), NPH (16) Gestational: glargine (15) NPH (44)	Pregestational Gestational	Insulin glargine versus NPH Pregestational versus gestational DM
[38]	Imbergamo et al. (2008)	Case control	January 2004 to December 2007	73	Glargine (15) NPH (15) Control (43)*	Type 1 DM	Insulin glargine versus NPH Insulin glargine versus control NPH versus control
[39]	Negrato et al. (2010)	Observational prospective	January 2004 to April 2009	138	Pregestational: glargine (18), NPH (38) Gestational: glargine (37), NPH (45)	Pregestational Gestational	Insulin glargine versus NPH Pregestational versus gestational DM
[40]	Poyhonen-Alho et al. (2007)	Case control	January 2003 to December 2005	91	Glargine (42) NPH (50)	Type 1 DM	Insulin glargine versus NPH
[41]	Price et al. (2007)	Case control	January 2002 to December 2005	64	Pregestational: glargine (10), NPH (10) Gestational: glargine (22), NPH (22)	Pregestational (type 1 DM) Gestational	Insulin glargine versus NPH Pregestational versus gestational DM
[42]	Smith et al. (2009)	Retrospective	January 2000 to December 2005	52	Glargine (27) NPH (25)	Pregestational (type 1+2 DM) Gestational	Insulin glargine versus NPH

*Control subjects were not included in the meta-analysis as they did not receive basal insulin treatment.

**Table 2:** Baseline maternal characteristics of pregnant women using insulin glargine versus NPH among the studies selected for meta-analysis.

Maternal characteristic	Number of studies	Insulin glargine		NPH		Mean difference*	95% confidence limits	
		Unadjusted mean/events	Number of Patients	Unadjusted mean/events	Number of Patients		Lower limit	Upper limit
Maternal age (yrs)	7	30.3	289	30.5	322	−0.49	−1.87	0.89
Duration of diabetes (yrs)	7	10.2	250	10.3	278	1.14	0.28	2.00
Type 1 diabetes	5	15.4	117	14.6	133	1.67	0.82	2.52
Pregestational diabetes	3	6.1	140	6.4	146	0.69	−0.96	2.35
Prepregnancy weight (kg)	4	82.0	154	80.1	190	1.53	−2.21	5.27
Prepregnancy BMI (kg/m^2^)	3	31.0	140	29.7	139	−0.46	−1.81	0.90

*Mean difference: insulin glargine versus NPH insulin.

**Table 3:** Maternal outcomes of pregnant women using insulin glargine versus NPH among the studies selected for meta-analysis.

Maternal outcomes	Number of studies	Insulin glargine		NPH		Mean difference*/ odds ratio**	95% confidence limits	
		Unadjusted mean/events	Number of patients	Unadjusted mean/events	Number of patients		Lower limit	Upper limit
Weight at delivery (kg)	4	93.3	167	92.1	179	−0.82*	−6.79	5.15
Weight gain (kg)	5	15.1	230	15	265	0.16*	−1.03	1.35
HbA_1c_—1st trimester (%)	4	7.67	143	7.65	158	−0.08*	−0.64	0.49
HbA_1c_—3rd trimester (%)	6	6.7	252	6.8	286	−0.01*	−0.07	0.05
Severe hypoglycemia (*n*)	4	9	155	20	205	0.84**	0.18	3.79
Pre-eclampsia (*n*)	8	26	331	40	371	0.55**	0.23	1.32
Cesarean section (*n*)	6	199	284	231	324	1.04**	0.72	1.52
Gestational hypertension (*n*)	4	9	155	23	205	0.49**	0.20	1.20

*Mean difference: insulin glargine versus NPH insulin; **Odds ratio: insulin glargine/NPH insulin.

**Table 4:** Neonatal outcomes of pregnant women using insulin glargine versus NPH among the studies selected for meta-analysis.

Neonatal outcome	Number of studies	Insulin glargine		NPH		Mean Difference*/ odds ratio**	95% confidence limits	
		Unadjusted mean/events	Number of patients	Unadjusted mean/events	Number of patients		Lower limit	Upper limit
Gestational age at delivery (wks)	7	37.3	289	37	322	0.09*	−0.43	0.61
Birth weight (g)	7	3463	288	3412	313	12.97*	−19.18	45.12
NICU admissions (*n*)	6	94	274	94	307	0.79**	0.45	1.38
Apgar score—5 minute (<7, *n*)	4	6	149	4	183	1.36**	0.26	7.06
Macrosomia (>4000 g, *n*)	4	37	157	39	198	1.20**	0.71	2.02
LGA (>90th percentile, *n*)	4	58	165	75	216	1.05**	0.68	1.63
Congenital malformations (*n*)	5	17	237	23	271	0.78**	0.39	1.59
Respiratory distress syndrome (*n*)	6	24	261	15	288	1.62**	0.82	3.21
Neonatal hypoglycemia (*n*)	7	58	304	62	346	0.99**	0.63	1.56
Hyperbilirubinemia (*n*)	6	58	272	60	314	0.93**	0.49	1.79

*Mean difference: insulin glargine versus NPH insulin; **Odds ratio: insulin glargine/NPH insulin.
